# Analysis of pulmonary artery variation based on 3D reconstruction of CT angiography

**DOI:** 10.3389/fphys.2023.1156513

**Published:** 2023-04-27

**Authors:** Xiaochao Ma, Tianyu Lu, Da Qin, Hongfei Cai, Ze Tang, Yue Yang, Youbin Cui, Rui Wang

**Affiliations:** ^1^ Department of Thoracic Surgery, The First Hospital of Jilin University, Changchun, Jilin, China; ^2^ School of Public Health, Jilin University, Changchun, Jilin, China

**Keywords:** pulmonary artery variation, three-dimensional reconstruction, CT angiography, preoperative assessment, surgical planning

## Abstract

**Objective:** The aim of this study is to acquire pulmonary CT (Computed tomography) angiographic data for the purpose of creating a three-dimensional reconstruction. Additionally, we aim to analyze the features and deviations of the branches in both pulmonary lobes. This information is intended to serve as a more comprehensive and detailed reference for medical professionals when conducting preoperative evaluations and devising surgical plans.

**Method:** Between August 2019 and December 2021, 420 patients were selected from the thoracic surgery department at the First Hospital of Jilin University, and underwent pulmonary 64 channel contrast enhanced CT examinations (Philips ICT 256). The images were acquired at a 1.5 mm slice thickness, and the DCM files that complied with DICOM (Digital Imaging and Communications in Medicine) standards were analysed for 3D (three dimensional) reconstruction using Mimics 22.0 software. The reconstructed pulmonary artery models were assessed by attending chest surgeons and radiologists with over 10 years of clinical experience. The two-dimensional image planes, as well as the coronary and sagittal planes, were utilized to evaluate the arteries. The study analyzed the characteristics and variations of the branches and courses of pulmonary arteries in each lobe of the lungs, with the exception of the subsegmental arterial system. Two chest surgeons and two radiologists with professional titles-all of whom had over a decade of clinical experience-jointly evaluated the 3D models of the pulmonary artery and similarly assessed the characteristics and variations of the branches and courses in each lobe of the lungs.

**Results:** Significant variations were observed in the left superior pulmonary artery across the 420 subjects studied. In the left upper lobe, the blood supply of 4 arteries accounted for 50.5% (*n* = 212), while the blood supply of 2 arteries in the left lower lobe was the most common, accounting for 79.5% (*n* = 334). The greatest variation in the right pulmonary artery was observed in the branch supply of the right upper lobe mediastinal artery. In the majority of cases (77.9%), there were two arteries present, which was the most common configuration observed accounting for 64% (*n* = 269). In the right inferior lobe of the lung, there were typically 2–4 arteries, with 2 arteries being the most common configuration (observed in 79% of cases, *n* = 332).

**Conclusion:** The three-dimensional reconstruction of pulmonary artery CT angiography enables clear observation of the branches and distribution of the pulmonary artery while also highlighting any variations. This technique holds significant clinical value for preoperative assessments regarding lesions and blood vessels.

## Introduction

In recent years, lung cancer incidence rates have rapidly increased, posing a significant threat to public safety (CHHIKARA and PARANG, 2023). For low-grade malignant lung tumors, early detection and surgical intervention can increase the likelihood of successful recovery and longer survival times. However, surgical resection of lung tumors carries a high risk of bleeding, particularly in cases involving variations in the pulmonary artery. Pulmonary artery rupture and hemorrhage can be rapid and massive, resulting in hemorrhagic shock, cardiac arrest, cerebral ischemia, and even brain death (MEI et al., 2017). While traditional open chest surgery has a wider field of vision and can be performed over a broader range, hemostasis measures such as compression and vascular suture are difficult to apply in the case of pulmonary hemorrhage. In contrast, thoracoscopy has increasingly become an attractive option for pulmonary surgery. (GU et al., 2018; QIN et al., 2019; JIANG et al., 2020; NATH et al., 2022). However, thoracoscopic surgery has a limited connection to the outside world, preventing timely measures such as vessel suturing in case of pulmonary artery rupture or hemorrhage. Consequently, chest opening and delayed rescue may increase bleeding, increase operative and postoperative complications, and overall mortality risk (TAKAHASHI et al., 2019). Pulmonary blood vessels have a very high anatomical complexity, and there are inevitably some errors in the evaluation of pulmonary blood vessels using traditional two-dimensional imaging analysis before surgery. Three-dimensional reconstruction of pulmonary blood vessel models can significantly reduce these errors (XU et al., 2023). Prior to surgery, a comprehensive assessment of pulmonary blood vessel orientation and variation, encompassing pulmonary veins, arteries, and each lobar bronchus, is paramount to reduce bleeding risks during surgery and to formulate effective preoperative surgical plans (ICHINOSE et al., 2019). Recent clinical studies suggest that sub-segmental resection of the lung is superior to traditional segmental resection in terms of fewer complications and similar prognoses, highlighting the importance of using three-dimensional reconstruction technology to improve preoperative pulmonary vascular assessment (FAN et al., 2022; SAJI et al., 2022). Therefore, obtaining an accurate understanding of the pulmonary artery’s orientation and variation before surgery is of significant value in reducing bleeding risks during surgery (NIA et al., 2019).

In recent years, computer technology has rapidly developed, and 3D reconstruction technology and artificial intelligence are the latest advancements (NYBERG et al., 2017; CAI et al., 2018; LOFTUS et al., 2020; LI et al., 2022; LI et al., 2023). The field of medicine has also benefited from this technology, resulting in the development of medical three-dimensional reconstruction technology. This advanced technology involves using CT, MRI (Magnetic resonance imaging), and other scanning techniques to collect continuous data of the body structure and store it in a computer. Relevant software is then used to recognize and process the image data, which is utilized to construct a virtual three-dimensional model of human organs (ZHOU et al., 2020; CHADWICK et al., 2021; KNUDSEN et al., 2021; WANG et al., 2023). Compared with two-dimensional images, the reconstructed three-dimensional model has the advantages of being more intuitive, easier to observe, and presenting a better understanding of the relevant tissues in the body. CT is an optimal scanning method, being characterized by thin layers, fast scanning speed, low cost, and easy data access. For example, the angiographic technique enables the injection of a contrast medium into the blood vessel, thus enabling better observation of the vessels’ different CT values from surrounding tissues (WANG et al., 2020). Using pulmonary CT angiography, computer DCM image data can be obtained, allowing for the convenient and straightforward reconstruction of the corresponding pulmonary artery model through inputting the data into relevant three-dimensional reconstruction software. Additionally, pulmonary artery variations can be analyzed, and the branch and course of the pulmonary artery can be observed clearly, which is far more convenient and easy than the traditional anatomical dye perfusion method (FAN et al., 2022).

The main objective of this study is to gather CT angiography data of the lung for three-dimensional reconstruction purposes. This data will be utilized to analyze the characteristics and the variation of the branches of bilateral pulmonary lobar arteries. Through this analysis, the safety of clinical operations related to the lungs will be improved, and the application value of three-dimensional reconstruction technology in pulmonary vascular reconstruction will be explored.

## Methods

This study was conducted on patients in the Department of Thoracic Surgery at No. 1 Hospital of Jilin University between August 2019 and December 2021. The ethics committee of the First Hospital of Jilin University approved this study, and the audit batch number assigned to this research was 2019-069.

### Inclusion criteria

1) Age (≥18 years old), age (<60 years old), sex is not limited.
2) Pulmonary CT angiography was performed in the Department of Radiology, First Hospital of Jilin University. No data were available.
3) There is no lesion or small lesion in both lungs, which does not affect the course of blood vessels.
4) Imaging data without history of thoracic trauma or operation;
5) The two-dimensional data of pulmonary CT angiography were well enhanced and the model was clear for three-dimensional reconstruction.

### Exclusion criteria

1) The pulmonary CT angiographic data were incomplete.
2) Pulmonary CT angiography in the outpatient hospital.
3) The pulmonary lesions are larger or there are factors affecting the course of pulmonary artery.
4) Two-dimensional data of pulmonary CT angiography showed no obvious enhancement of arteries, and the model was not clear after three-dimensional reconstruction, which affected observation and statistics.

### Standardized operation process

For the pulmonary CT angiography performed at No. 1 Hospital of Jilin University, patients in the radiology department were subjected to peripheral intravenous injection of iodinated contrast agents. After approximately 70 s, the lung CT continuous scan commenced, following which the data was stored in DCM format files for further analysis. In this study, data reconstruction was performed using the Mimics 22.0 three-dimensional (cck: 8A21-3B97-D4C7-9984) reconstruction software.

**FIGURE 1:**
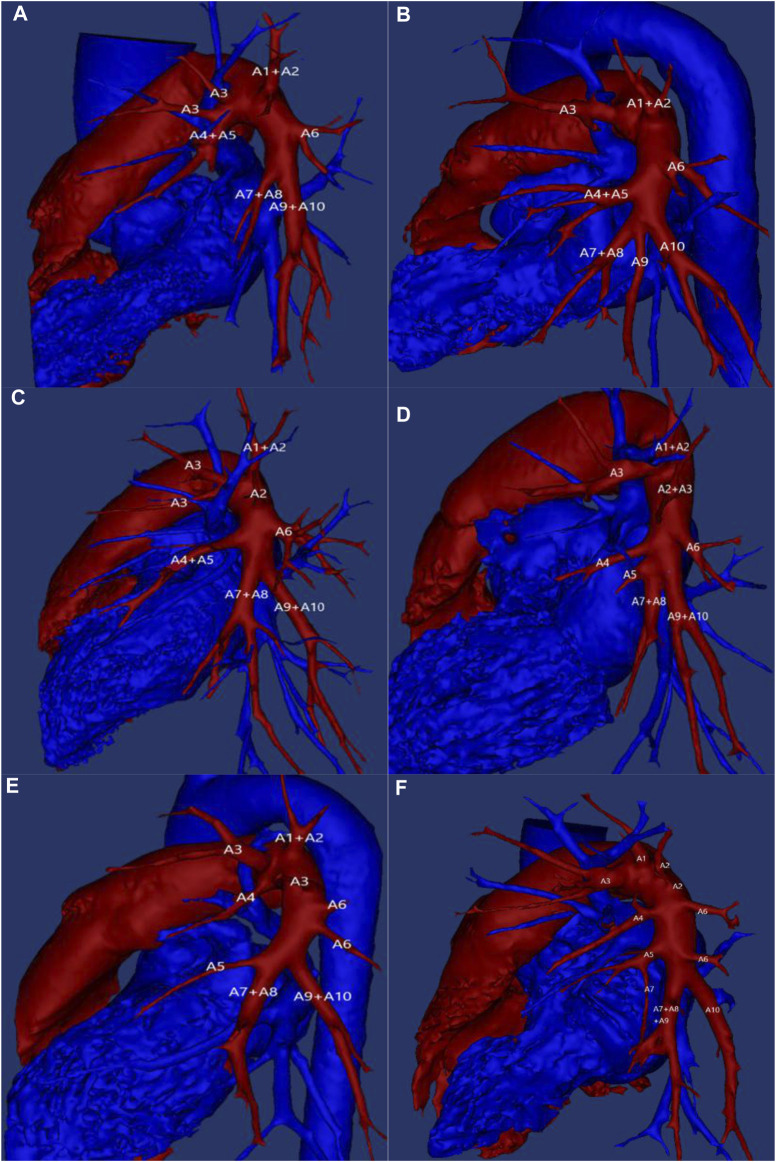
Example of left pulmonary artery vascular variation. A1: Apical segmental artery; A2: Posterior segmental artery; A3: Anterior segmental artery; A4: Superior lingual segmental artery; A5: Inferior lingual segmental artery; A6: Inferior lobe superior segment artery; A7: Medial basal segment artery; A8: Anterior basal segment artery; A9: lateral basal segment artery; A10: posterior basal segment artery.

**FIGURE 2:**
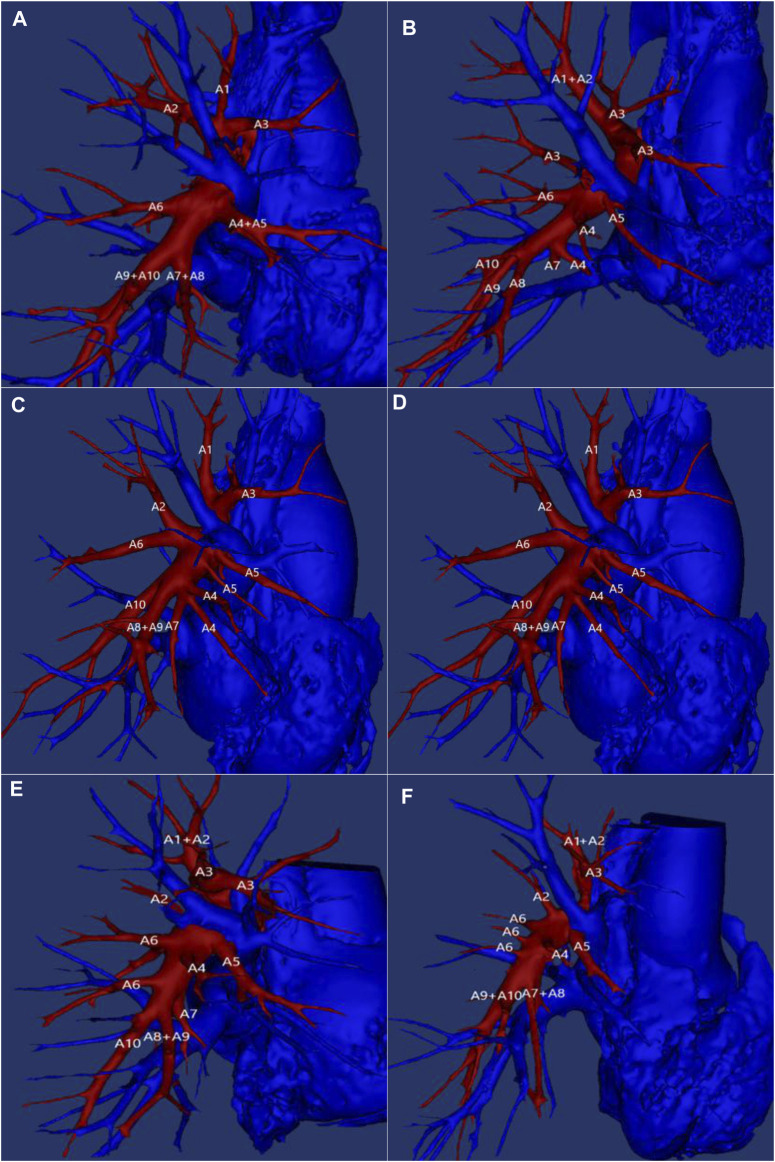
Example of right pulmonary artery vascular variation. A1: Apical segmental artery; A2: Posterior segmental artery; A3: Anterior segmental artery; A4: Lateral segmental artery of middle lobe; A5: Medial segmental artery of middle lobe; A6: Inferior lobe superior segment artery; A7: Medial basal segment artery; A8: Anterior basal segment artery; A9: lateral basal segment artery; A10: posterior basal segment artery.

1. Import the image file with Mimics 22.0.
2. The two-dimensional CT angiography images of the lung were reconstructed after the introduction.
3. After that, the target artery is segmented into surrounding tissues by “Thresholding,” and then the model (Figures 1, 2) is obtained by using “Dynamic Region Growing” and “Calculate 3D.”
4. Following the reconstruction, the three-dimensional model of the pulmonary artery was assessed collaboratively by two chest surgeons and two radiologists, all holding professional titles and possessing over 10 years of clinical experience. They meticulously scrutinized and tallied the pulmonary vascular course and branch variations, and subjected their observations to thorough scrutiny and reconciliation through double entry.

### Major instruments and equipment

DISCOVERY TM 64 CT (Philip, ICT 256, German) were used for high resolution (64-spiral) enhanced chest CT, MIMICS 22.0 (Belgium Materialize company) for Three-dimensional reconstruction. Position of patients: head forward, supine position, hands up to head.

### Statistical method

In the present study, IBM SPSS 19.0 was utilized to perform statistical analysis on all acquired data. Continuous variables that exhibited normal distribution were represented using Mean ± SD, while non-normally distributed continuous variables were expressed as M (P25, P75). Group comparison of means was conducted using *t*-test, whereas variance analysis was employed for inter-group comparisons. The chi-squared test was employed to unveil distributional differences. Statistical significance was set at *p* < 0.05, indicating a meaningful statistical difference.

## Result

This study involved 420 subjects, comprising of 216 males and 204 females. Table 1 displays that the average age of the subjects was 56.54 ± 10.08 for males and 56.12 ± 9.55 for females.

**TABLE 1:** Basic data of all the subjects.

Characters	Sex groups		*t*	*p*
	Male (*n* = 216)	Female (*n* = 204)		
Age	56.54 ± 10.08	56.12 ± 9.55	0.44	0.66

We conducted three-dimensional reconstruction of the pulmonary vessels using enhanced CT images of the lungs in all participants. We identified two variants of the pulmonary artery as follows.

### Variation of left superior pulmonary artery

According to the three-dimensional reconstruction results, 420 subjects in this study had a significant degree of variation in their left superior pulmonary artery as Table 2 shows. The number of blood supply arteries varied between 2–7, of which 2 arteries constituted 1.4% (*n* = 6) (Figure 1A), 3 arteries were found in 25.5% (*n* = 107) of the cases (Figure 1B), 4 arteries were present in 50.5% (*n* = 212) of the cases (Figure 1C), 5 arteries were present in 21.6% (*n* = 91) of the cases (Figure 1D), while 6 arteries were present in only 0.5% (*n* = 2) (Figure 1F). Additionally, 7 arteries were found in only 0.5% (*n* = 2) of the cases. Four arteries were the most common blood supply to the upper left lung, followed by three and five arteries. On the other hand, two and six or seven arteries provided lesser supply.

**TABLE 2:** Characteristics of left superior pulmonary artery.

Number of arteries	Groups		χ^*2*^	*p*
	Male	Female		
2 arteries (*n* = 6.1.4%)	0	6	8.146	0.228
3 arteries (*n* = 107.25.5%)	56	51		
4 arteries (*n* = 212.50.5%)	109	103		
5 arteries (*n* = 91.21.6%)	51	40		
6 arteries (*n* = 2.0.5%)	0	2		
7 arteries (*n* = 2.0.5%)	0	2		

### Left inferior lobar artery variation

The left inferior lobar pulmonary artery constitutes the terminal portion of the left pulmonary artery, continuing to extend downward after the separation of the left superior lobar pulmonary artery. For the purpose of this study, the left inferior lobe basilar artery was also considered as an artery (Table 3). The left inferior lobe is known to contain 2–4 arteries, with the number and distribution of arteries varying in the upper segment of the left inferior lobe. Among the 420 subjects enrolled in this study, 79.5% (*n* = 334) presented with one superior artery + one basilar artery (Figure 1A), 19.5% (*n* = 82) with two superior arteries + one basilar artery (Figure 1E), and 1% (*n* = 4) with three superior arteries + one basilar artery. It was found that the left inferior lobe primarily comprised a single superior-inferior lobar artery + one basilar artery trunk, while the basal segment of the inferior lobe received 2, 3 or 4 basilar arteries based on individual variations.

**TABLE 3:** Distribution characteristics of left inferior pulmonary artery.

Left inferior lobar artery	*N*	%
2 arteries supply blood, --1 upper segment artery	334	79.50
3 arteries supply blood, --2 upper segment arteries	82	19.50
4 arteries supply blood, --3 upper segment arteries	4	1.00

### Variation of right superior lobar pulmonary artery

The first artery of the right upper lobe of the lung typically originates from the mediastinal part of the right pulmonary artery, earning the name of the mediastinal artery. Its supply artery may consist of 1–4 branches (Table 4). The findings showed that one artery, specifically the mediastinal artery, supplied the upper lobe tip, posterior, and anterior segments, accounting for 7.6% (*n* = 32), as illustrated in Figure 2A. Two arteries, one mediastinal artery and an additional artery supplying the upper or lower segments, accounted for 77.9% (*n* = 327) as demonstrated in Figure 2B. Three arteries, comprising one mediastinal artery and two additional arteries supplying the upper or lower segments, accounted for 14% (*n* = 59). Four arteries, which included one mediastinal artery and three additional arteries, accounted for only 0.5% (*n* = 2).

**TABLE 4:** Distribution characteristics of the right superior lobar pulmonary artery.

Basilar artery stem from basilar artery	*N*	%
1 artery supplies blood	32	7.6
2 arteries supplies blood	327	77.9
3 arteries supplies blood	59	14.0
4 arteries supplies blood	2	0.5

### Variation of right middle pulmonary artery

The right middle pulmonary artery usually originates from the interlobar segment of the right pulmonary artery and extends towards the medial and lateral segments of the right middle pulmonary artery. The middle lobe can receive blood supply from 1–4 arteries. The first option is a large middle lobe artery trunk which divides into medial and lateral arteries, which accounts for 30.5% of researchers (*n* = 128), as shown in Figure 2A. The second option is two middle lobe arteries from the right pulmonary artery, where the upper branch supplies the medial while the lower branch supplies the lateral segments. This accounts for 64% (*n* = 269) as shown in Figure 2B. The third choice is three middle lobe arteries accounting for 5% (*n* = 21) shown in Figure 2C. Lastly, four middle lobe arteries supply only 0.5% (*n* = 2) as shown in Figure 2D. Based on Table 5, it can be observed that two arteries are the most common, followed by one middle lobe artery as the second most common, while three and four arteries are less frequent.

**TABLE 5:** Variation of right superior lobar pulmonary artery.

Number of right middle pulmonary artery		Location of artery				
		Between the mediastinal artery of the upper lobe and the superior artery of the lower lobe	Level of right inferior lobar superior pulmonary artery	Between superior inferior lobar artery and basilar artery trunk	Bifurcation of basilar artery	Under the bifurcation of the basilar artery trunk
1middle lobe artery (*n* = 128, 30.5%)		128 (100.0%)	0 (0.0%)	0 (0.0%)	0 (0.0%)	0 (0.0%)
2 middle lobe arteries (*n* = 269, 64%)	1	258 (96.0%)	11 (4.0%)	0 (0.0%)	0 (0.0%)	0 (0.0%)
	2	6 (2.2%)	118 (43.9%)	122 (45.4%)	21 (7.8%)	2 (0.7%)
3 middle lobe arteries (*n* = 21, 5%)	1	21 (100%)	0 (0.0%)	0 (0.0%)	0 (0%)	0 (0.0%)
	2	0 (0.0%)	8 (38.1%)	11 (52.4%)	2 (9.5%)	0 (0.0%)
	3	0 (0.0%)	0 (0.0%)	6 (28.6%)	15 (71.4%)	0 (0.0%)
4 middle lobe arteries (*n* = 2, 0.5%)	1	2 (100.0%)	0 (0.0%)	0 (0.0%)	0 (0%.0)	0 (0.0%)
	2	0 (0.0%)	2 (100%)	0 (0.0%)	0 (0.0%)	0 (0.0%)
	3	0 (0.0%)	0 (0.0%)	0 (0.0%)	2 (100%)	0 (0.0%)
	4	0 (0.0%)	0 (0.0%)	0 (0.0%)	0 (0.0%)	2 (100.0%)

### Variation of right inferior lobar pulmonary artery

The right inferior lobar pulmonary artery is formed by the extension of the right pulmonary artery from the middle lobe artery. This formation is described in Table 6 where in the right inferior lobe can comprise 2–4 arteries when taking the right inferior lobe artery as the main artery. The main variation in these arteries is observed in the number of upper segment arteries. As per the findings of Table 5, 79% of cases (*n* = 332) had one upper segment artery along with one basilar artery trunk (Figure 2A). In 20.5% cases (*n* = 86), two upper segment arteries along with one basilar artery trunk were observed (Figure 2E). Meanwhile, only 0.5% of cases (*n* = 2) had three upper segment arteries along with one basilar artery trunk (Figure 2F). Hence, for the right lower lobe of the lung, the blood supply is mainly through 2 arteries followed by 3 arteries, and 4 arteries are seldom seen.

**TABLE 6:** The vascular characteristics of the right inferior lobar pulmonary artery.

Number of arterial blood supply	*N*	%
2 arteries supply blood (1 upper segment artery)	332	79.0
3 arteries supply blood (2 upper segment arteries)	86	20.5
4 arteries supply blood (3 upper segment arteries)	2	0.5

### The clinical application of three-dimensional reconstruction

**FIGURE 3:**
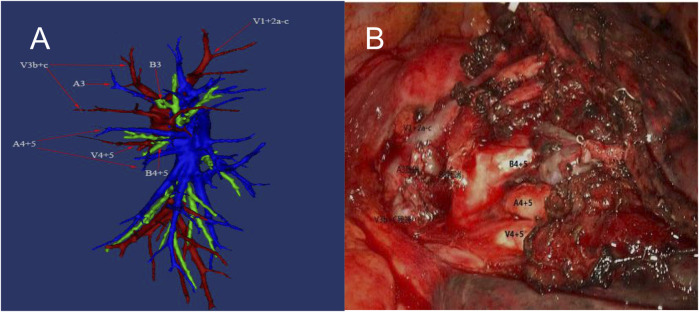
Preoperative reconstruction (left) and intraoperative vascular deformation. A3: Anterior segmental artery; A4: Superior lingual segmental artery; A5: Inferior lingual segmental artery; B3: Anterior segmental bronchus; B4: superior lingual segmental bronchus. B5: Inferior lingual segmental bronchus; V1 + V2a–c: Apical segmental vein + Posterior segmental vein and Its branches; V3b + c: Two branches of anterior segmental vein; V4 + V5: Superior lingual segmental vein + Inferior lingual segmental vein.

We have conducted a small amount of research to continue exploring the practical benefits of applying the reconstructed arterial model to the surgical process. Our findings have further elucidated the clinical significance of three-dimensional pulmonary artery reconstruction (as Figure 3 shows). However, due to the limited scope of our study, which only covered a small number of clinical cases at a single center, its relevance is still only of reference significance for the time being. Nevertheless, this study has provided a valuable direction for future research aimed at fully understanding the utility of this technology in a wider variety of clinical settings.

## Discussion

In this study, the left superior pulmonary artery system displayed the highest degree of variation among both pulmonary arteries (Maki et al., 1007). Cory and Valentine surveyed anatomical variations in the pulmonary artery tree in 426 patients between 1955 and 1959, among whom 513 underwent anatomical pneumonectomy. The pair analyzed 524 pulmonary lobes or pulmonary arterial and vascular systems and documented 29 variant types of arteries through 107 resections of the left upper lobe of the lung. They determined that there were almost no authentic arterial types in the left upper lobe of the lung and that the number of arteries ranged from 2 to 7, which was consistent with the results presented in Table 2 of this study. In this study, we documented 43 types of arteries in 200 cases of left superior pulmonary artery three-dimensional reconstruction. The total number of types 3, 5, 10, and 13 accounted for 42.5% (*n* = 85), which was in line with Cory and Valentine’s findings. Maciejewski (MACIEJEWSKI and KUTNIK, 1990) studied 100 pairs of stained, injected, or corroded cast lungs in 1900 and described the characteristics of the left superior pulmonary artery. He concluded that the left superior pulmonary artery was mostly supplied by four arteries (49%). Our results in Table 3 were consistent with this finding (50.5%). Fourdrain (FOURDRAIN et al., 2018) used CT angiography to perform three-dimensional reconstruction of the arteries in 44 surgically operated patients and found that the lingual segmental artery was supplied via two common arteries (77.3%), which was different from our findings (61.5%), and by two separate lingual arteries (37%). We also identified three lingual arteries (*n* = 3). As per our observations, the lingual artery (24%) with mediastinal origin (originating from the anterolateral wall of the first artery in the left upper lobe of the lung) was more common than Fourdrain’s (15.9%), and some studies have reported that the lingular segment was dispatched before the proper segment artery and had an incidence rate of about 5.6% (GAO et al., 2021). The lingular artery was identified as the only source for the lingual segment, accounting for 11% of the total supply, which is a significant difference from Fourdrain’s previous findings of only 2.3%.

Compared to the left upper lobe, the arterial system of the left inferior lobe is less complex. Previous studies by Cory and Valentine (CORY and VALENTINE, 1959) have reported four main types of arteries in the left lower lobe of the lung, including one apical segment artery and two common trunk basilar arteries. However, in our study, we identified a total of eight types of arteries in the left lower lobe. Notably, we observed that four basilar arteries (*n* = 2) could be separated from the left lower lobe basilar artery trunk and extended from the upper lingual artery to the basilar artery (*n* = 1).

In Fourdrain’s previous study (FOURDRAIN et al., 2018), one superior segment artery was observed in 65.9% cases, while two and three superior segments were present in 27.3% and 6.8% cases, respectively. In our study, we found that the frequency of one superior segment artery (79.5%) was higher than that reported by Fourdrain. On the other hand, the frequencies of two (19.5%) and three (1%) superior arteries were lower than those observed by Fourdrain. With regard to the basilar artery, we identified two common trunks (anterolateral and posterolateral trunks) accounting for 52.5%, which is similar to Fourdrain’s findings (50%). However, we observed a lower incidence of three basilar arteries (17%) compared to Fourdrain’s observation (50%).

In this study, we utilized the Cabrol and Corner terminology to describe the arterial system of the right superior lobe. Typically, the first branch of the right pulmonary artery is known as the mediastinal artery of the right upper lobe. It originates exclusively from the mediastinum due to the longer path of the right pulmonary artery originating from the left ventricle. The French Encyclopedia of Surgery describes the right pulmonary artery tree as consisting of one mediastinal artery, one or two middle lobe arteries, and the upper and basal segments of the right lower lobe (which is divided into four segments). Murota (MUROTA et al., 2015) utilized three-dimensional reconstruction of pulmonary artery tree and MPR imaging to achieve high identification rates of right superior pulmonary artery branches, which were 97.2% and 99.7%, respectively.

Our study identified a total of 24 types of arteries in the right upper lobe of the lung. However, two types of arteries, namely, the mediastinal artery and dorsal mediastinal artery, accounted for 78% of cases. The main difference between the different types of arteries was the various segments of the mediastinal artery branches. Apart from the mediastinal artery, 78% (*n* = 156) of cases had an additional artery, which was similar to Fourdrain’s finding of 70.5%. A few cases had two or three additional arteries. Our study also revealed that the superior lobe artery originating from the dorsal side of the plane of the right middle lobe could simultaneously supply both anterior, posterior or bifurcated segments. Moreover, we observed the co-trunking of the superior right lower lobe artery with this artery.

### Right middle lobe pulmonary artery

The supply of the right middle lobe artery was relatively fixed, similar to that described by Fourdrain and Corey and Valentine, with two segmental middle lobe arteries or a common middle lobe artery. Cory and Valentine found five types of arteries in the right middle lobe of the lung, of which 49% (*n* = 25) were supplied by two middle lobe arteries and 45% (*n* = 23) were supplied by one single middle lobe artery. Six types of arteries were observed in the middle lobe of the right lung. Two middle lobe arteries (64%) were more frequently supplied than Cory and Fourdrain (54.5%). The supply of one middle lobe artery (30.5%) was less than that of Cory and Fourdrain (45.5%). We also observed three middle lobe arteries (*n* = 10) and the middle lobe arteries (*n* = 1) from the basilar artery, which were consistent with the findings of Cory and Valentine.

### Right inferior pulmonary artery

The right inferior pulmonary lobe artery is typically divided into the upper segment artery and basilar artery, with variations in the number of vessels observed between different types. Cory and Valentine previously identified six types of arteries, while in our study we observed seven types. Non-etheless, the vasculature of the right lower lobe bears similarities to a superior inferior lobe artery and a basilar artery trunk in most cases, consisting typically of two or three basilar arteries separated. Comparing basal segment vasculature across different anatomical studies can prove challenging, with variations in the level of artery division observed. Some studies have identified a gradual “dendritic” division, whereas others (MACIEJEWSKI, 1991; MACIEJEWSKI, 1992) have noted a “dense” division of the four segmental arteries. Our study found that 37% (*n* = 74) and 38% (*n* = 76) of the right inferior lobe basilar artery trunk featured an anterior internal basilar artery trunk and posterolateral basilar artery trunk, which aligns with Fourdrain’s previous findings. We additionally observed a modest 16.5% (*n* = 33) rate of dense or progressive dendritic division. Notably, our study identified further variations in the form of arteries extending from the superior segment to the basal segment (*n* = 9), as well as the presence of a superior inferior segment artery and the superior segment artery located at the back of the middle lobe artery plane (*n* = 8). We also identified additional posterior segment arteries (*n* = 9) at the bifurcation of the basal trunk, with four types of basilar artery present. Our findings indicate that when more than two upper segment arteries are present in the right lower lobe, the second upper segment arteries tend to emit beyond the bifurcation of the basal trunk (51.2%).

Despite our efforts to minimize errors through standardized operations and strict physician observation, our research is not without limitations. Firstly, the fact that our study only utilized single-center samples may lack the representativeness of corresponding multi-center studies. Secondly, the relatively small number of samples used in our research may have resulted in the amplification of corresponding errors. As such, further research utilizing larger samples and multi-center studies is needed in order to more fully investigate the topic at hand.

## Conclusion

The use of three-dimensional reconstruction of pulmonary artery CT angiography allows for more accurate observation of pulmonary artery branch characteristics and distribution, thus facilitating the identification of variations within the structure. Among the various lobes of both lungs, the left superior lobe artery displays the most striking variation and complexity.

## Data Availability

The original contributions presented in the study are included in the article/Supplementary Material, further inquiries can be directed to the corresponding authors.
