# Supplementary material for: Analysis of pulmonary artery variation based on 3D reconstruction of CT angiography
Source: Front Physiol. 2023 Apr 27;14:1156513. doi: 10.3389/fphys.2023.1156513 (PMC10206427; doi:10.3389/fphys.2023.1156513)
Supplement: Supplementary file 1 [file Table1.DOCX]

**
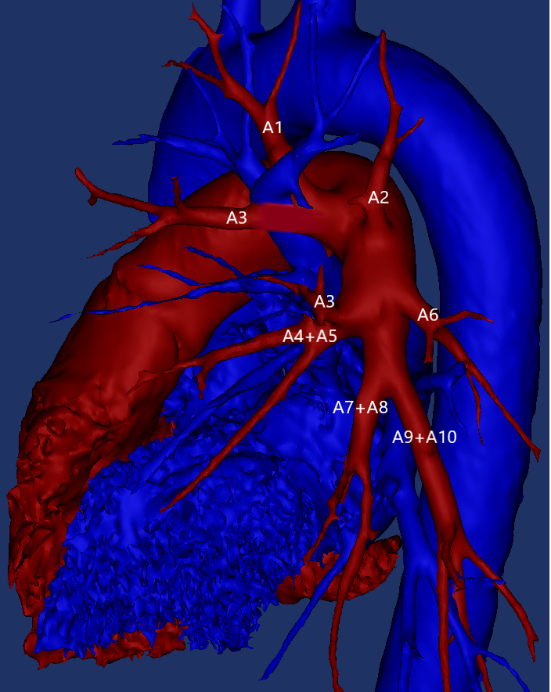

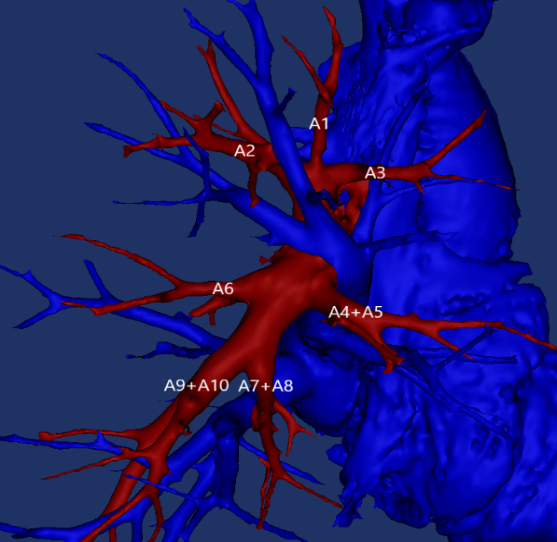

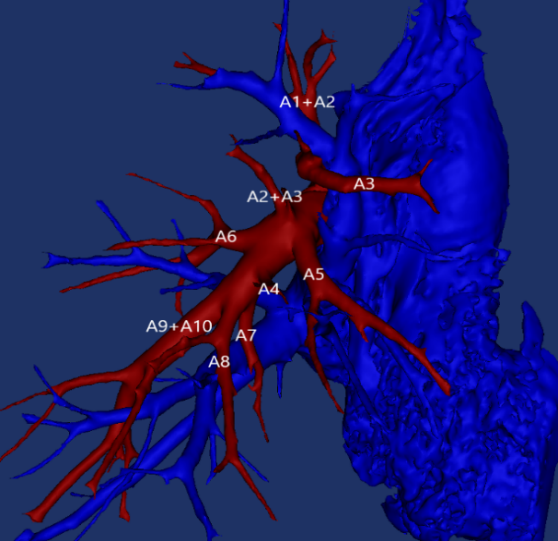

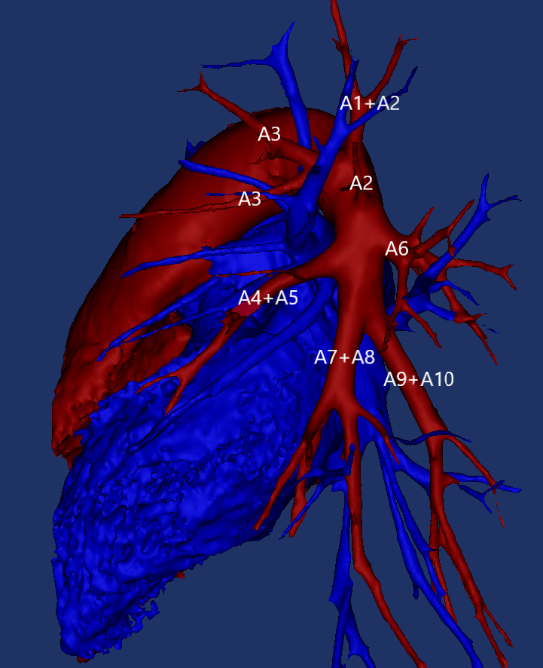
**

Figure 3：Left superior lobe of lung：A1：Apical segmental artery，A2：Posterior segmental artery，A3：Anterior segmental artery，A4+A5：Upper lingual segment and inferior lingual artery，

Left lower lobe of lung：A6：Inferior lobe superior segment artery，A7+A8：Trunk of anterior and medial basilar artery，A9+A10：Trunk of lateral and posterior basilar artery。

Figure 4：Right superior lobe of lung：A1：Apical segmental artery，A2：Posterior segmental artery，A3：Anterior segmental artery（A1、A2、A3 are all originate from the mediastinal artery）。

Middle lobe of right lung：A4+A5：Middle Lobar Artery (dividing into medial and lateral segmental arteries)

Right lower lobe of lung：A6：Inferior lobe superior segment artery，A7+A8：Trunk of anterior and medial basilar artery，A9+A10：Trunk of lateral and posterior basilar artery。

Figure 2: Right superior lobe of lung：A1+A2：Posterior apical artery ，A3：Anterior segmental artery（A1+A2 and A3 have the same trunk），A2+A3：The same trunk of posterior and Anterior segmental artery。

Middle lobe of right lung: Lateral segmental artery of middle lobe,A5:Medial segmental artery of middle lobe。

Right lower lobe of lung：A6：Inferior lobe superior segment artery，A7：Medial basal segment artery，A8：Anterior basilar segment artery，A9+A10：Trunk of lateral and posterior basilar artery。

Figure 1：Left superior lobe of lung：A3：Anterior segmental artery，A1+A2：Posterior apical artery，A2：Posterior segmental artery，A4+A5：The same trunk of upper lingual segment and Inferior lingual artery。

Left lower lobe of lung：A6：Inferior lobe superior segment artery，A7+A8：Trunk of anterior and medial basilar artery，A9+A10：Trunk of lateral and posterior basilar artery。


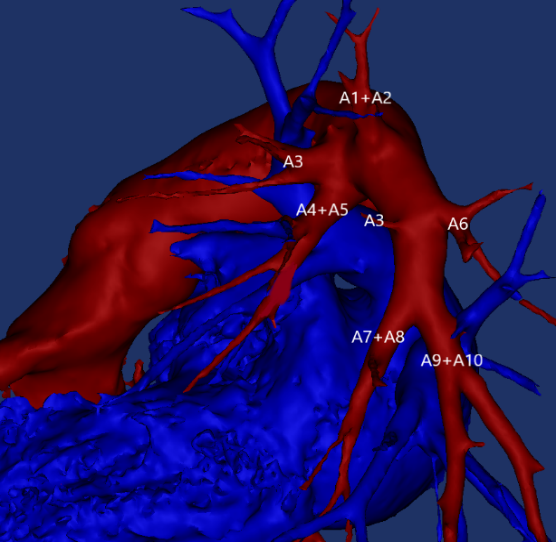

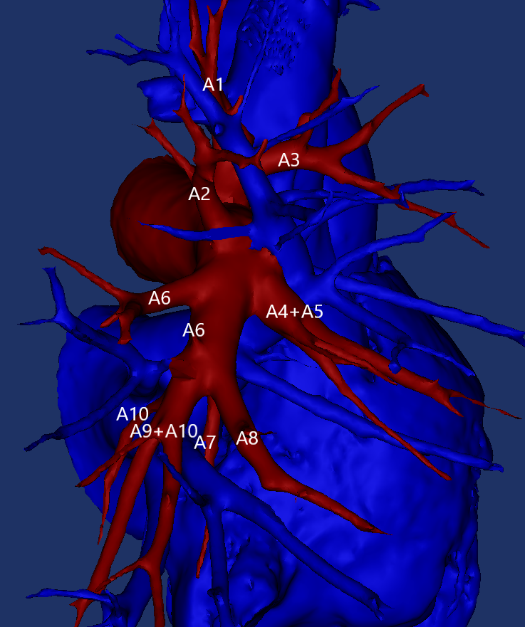

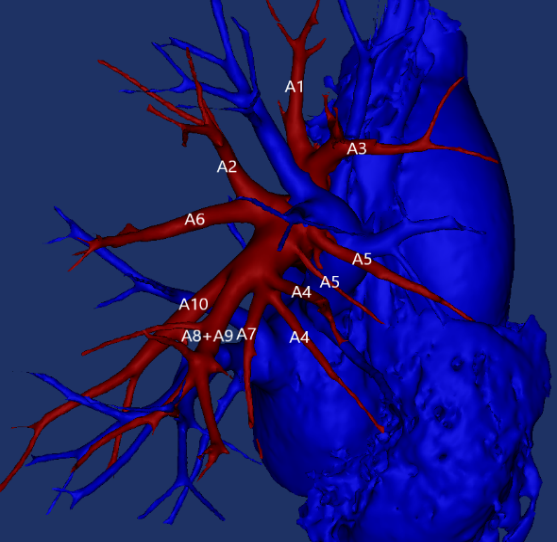

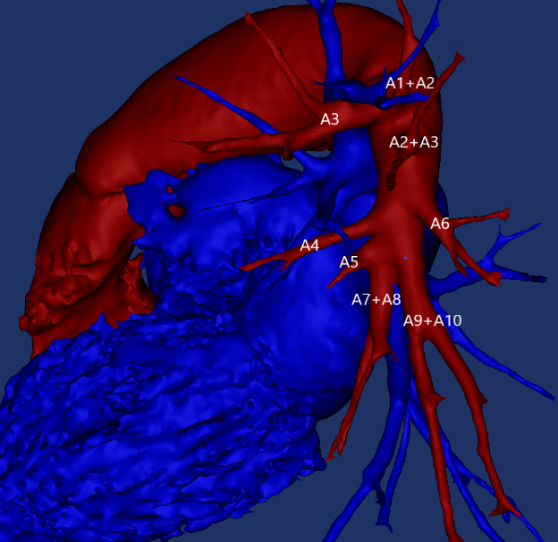


Figure 8：Right superior lobe of lung：A1：Apical segmental artery，A3：Anterior segmental artery（A1、A3 are both originate from the mediastinal artery），A1+A2:Apical segmental artery + Posterior segmental artery（have the same trunk）。

Middle lobe of right lung：A4+A5：Middle Lobar Artery (dividing into medial and lateral segmental arteries)。

Right lower lobe of lung：A6: Inferior lobe superior segment artery，A7：Medial basal segment artery，A8：Anterior basilar segment artery，A9+A10：Trunk of lateral and posterior basilar artery，A10：Posterior basilar segment artery。

Figure 7：Left superior lobe of lung：A1+A2：Posterior apical artery，A3：Anterior segmental artery，A4+A5：Superior lingual artery + Inferior lingual artery have the same trunk。

Left lower lobe of lung：A6：Inferior lobe superior segment artery，A7+A8：Trunk of anterior and medial basilar artery，A9+A10：Trunk of lateral and posterior basilar artery。

Figure 6：Right superior lobe of lung：A1：Apical segmental artery，A3：Anterior segmental artery（A1、A3 are both originate from the mediastinal artery），A2：Posterior segmental artery（The right lower lobe of lung upper segmental and superior lobe posterior segmental artery have the same trunk）。

Middle lobe of right lung：A4:Lateral segmental artery of middle lobe,A5:Medial segmental artery of middle lobe。

Right lower lobe of lung：A6：Inferior lobe superior segment artery，A7：Medial basal segment artery，A8+A9：Anterior and lateral basilar artery trunk，A10：Posterior basilar segment artery。

Figure 5：Left superior lobe of lung：A3：Anterior segmental artery，A1+A2：Posterior apical artery，A2+A3：posterior segmental and Anterior segmental artery，A4：Superior lingual artery；A5：Inferior lingual artery。

Left lower lobe of lung：A6：Inferior lobe superior segment artery，A7+A8：Trunk of anterior and medial basilar artery，A9+A10：Trunk of lateral and posterior basilar artery。


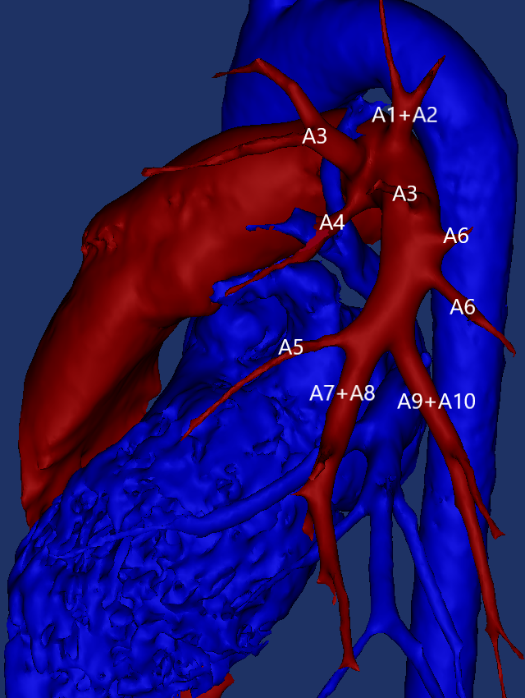

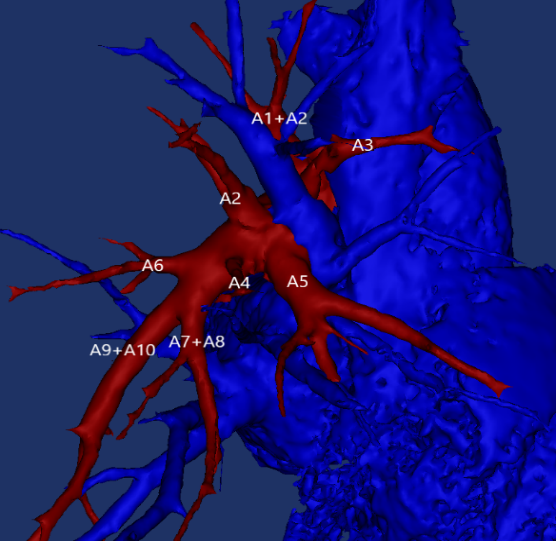

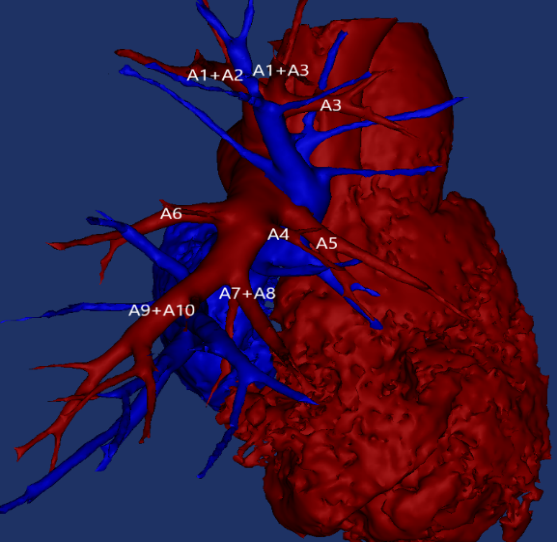

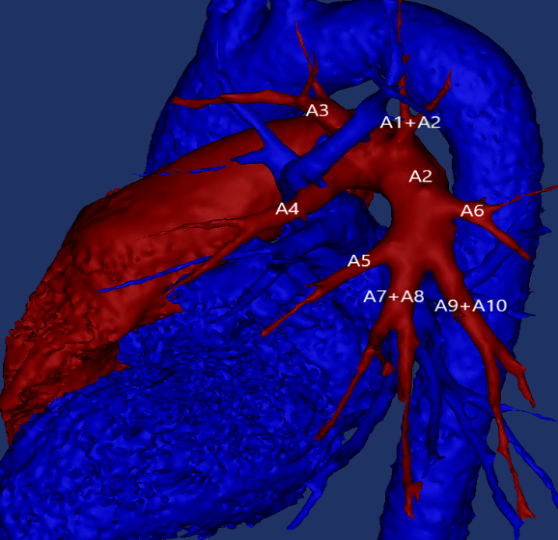


Figure 11：Left superior lobe of lung：A3：Anterior segmental artery，A1+A2：Posterior apical artery，A3：Anterior segmental artery，A4：Superior lingual artery，A5：Inferior lingual artery。

Left lower lobe of lung：A6：Inferior lobe superior segment artery，A7+A8：Trunk of anterior and medial basilar artery，A9+A10：Trunk of lateral and posterior basilar artery。

Figure 12：Right superior lobe of lung：A1+A2：Posterior apical artery，A3：Anterior segmental artery，A2：Posterior segmental artery。

Middle lobe of right lung：A4:Lateral segmental artery of middle lobe,A5:Medial segmental artery of middle lobe。

Right lower lobe of lung：A6：Inferior lobe superior segment artery，A7+A8：Trunk of anterior and medial basilar artery，A9+A10：Trunk of lateral and posterior basilar artery。

Figure 10：Right superior lobe of lung：A1+A2：Posterior apical artery，A1+A3：Apical artery + anterior segmental artery，A3：Anterior segmental artery（3 branches are all originate from the mediastinal artery）。

Middle lobe of right lung：A4:Lateral segmental artery of middle lobe,A5:Medial segmental artery of middle lobe。

Right lower lobe of lung：A6：Inferior lobe superior segment artery，A7+A8：Trunk of anterior and medial basilar artery，A9+A10：Trunk of lateral and posterior basilar artery。

Figure 9：Left superior lobe of lung：A3：Anterior segmental artery，A1+A2：Posterior apical artery，A2：Posterior segmental artery，A4：Superior lingual artery，A5：Inferior lingual artery。

Left lower lobe of lung：A6：Inferior lobe superior segment artery，A7+A8：Trunk of anterior and medial basilar artery，A9+A10：Trunk of lateral and posterior basilar artery。


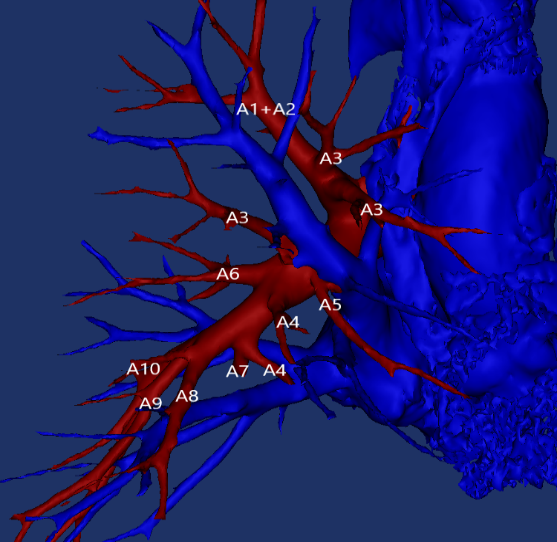

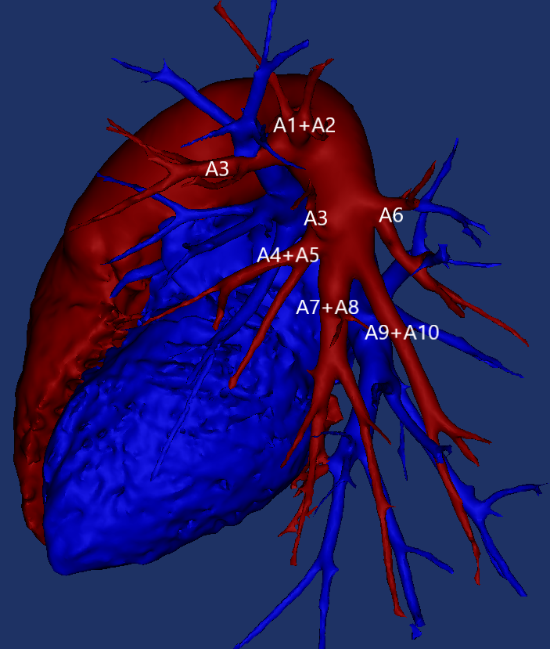

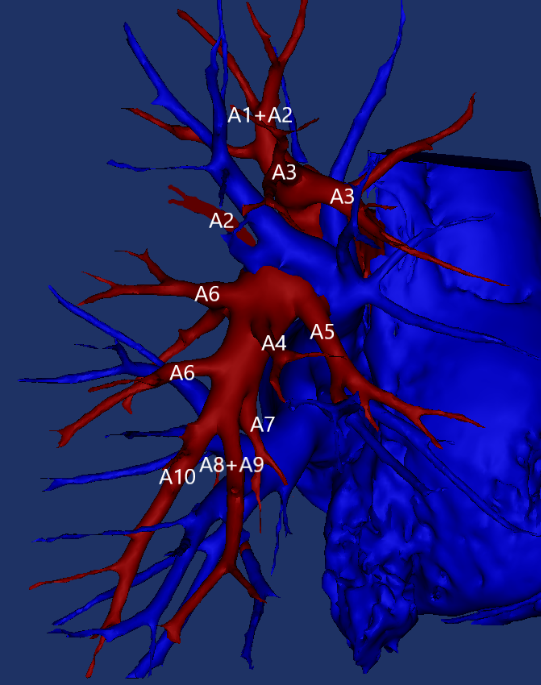

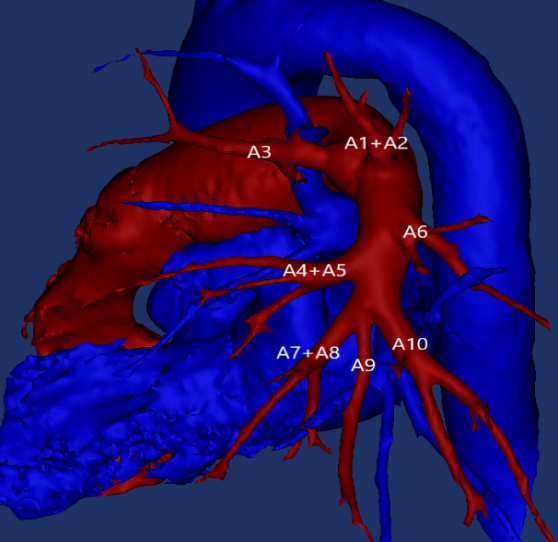


Figure 16:Right superior lobe of lung：A1+A2：Posterior apical artery，A3：Anterior segmental artery（A1+A2 and two branches of A3 are all originate from the mediastinal artery），A3：Anterior segmental artery。

Middle lobe of right lung：A4：Lateral segmental artery of middle lobe（The second A4 is originate from medial basal segment artery）,A5：Medial segmental artery of middle lobe。

Right lower lobe of lung：A6: Inferior lobe superior segment artery，A7：Medial basal segment artery，A8：Anterior basilar segment artery，A9：Lateral basal segment artery，A10：Posterior basilar segment artery。

Figure 15：Left superior lobe of lung：A3：Anterior segmental artery，A1+A2：Posterior apical artery，A4+A5：Superior lingual artery + Inferior lingual artery have the same trunk。

Left lower lobe of lung：A6：Inferior lobe superior segment artery，A7+A8：Trunk of anterior and medial basilar artery，A9+A10：Trunk of lateral and posterior basilar artery。

Figure 13：Left superior lobe of lung：A3：Anterior segmental artery，A1+A2：Posterior apical artery，A4+A5：Superior lingual artery + Inferior lingual artery have the same trunk。

Left lower lobe of lung：A6：Inferior lobe superior segment artery，A7+A8：Trunk of anterior and medial basilar artery，A9：Lateral basal segment artery，A10：Posterior basilar segment artery。

Figure 14:Right superior lobe of lung：A1+A2：Posterior apical artery，A3：Anterior segmental artery（A1+A2 and two branches of A3 are all originate from the mediastinal artery），A2：Posterior segmental artery。

Middle lobe of right lung：A4:Lateral segmental artery of middle lobe,A5:Medial segmental artery of middle lobe。

Right lower lobe of lung：A6: Inferior lobe superior segment artery，A7：Medial basal segment artery，A8+A9：Anterior and lateral basilar artery trunk，A10：Posterior basilar segment artery。


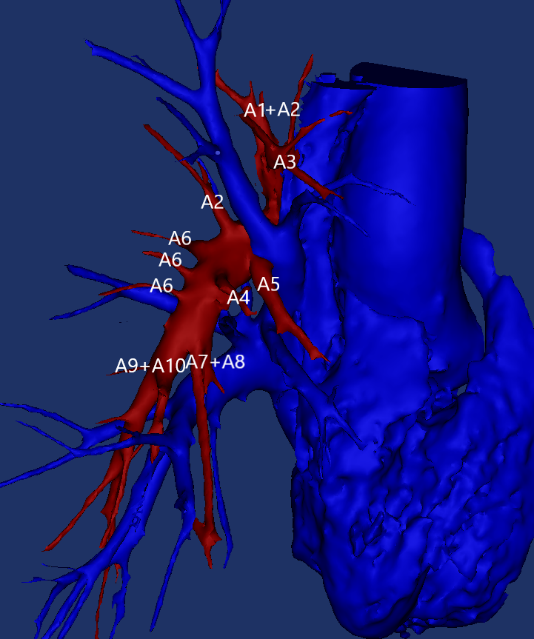

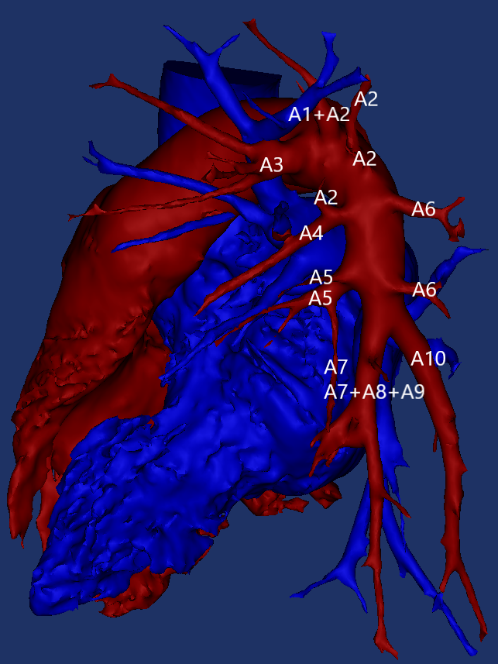

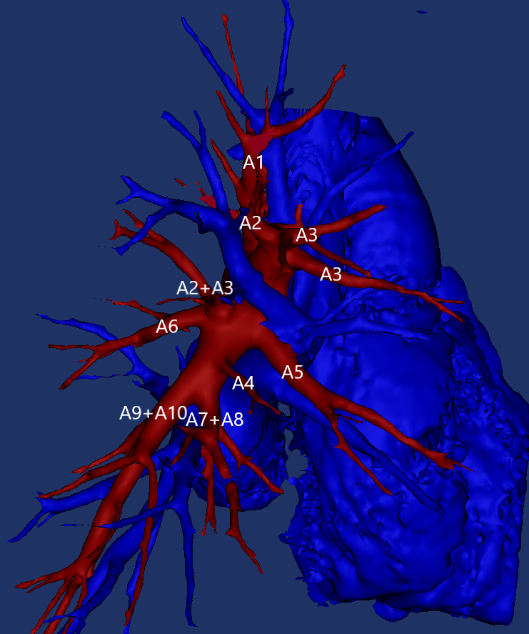

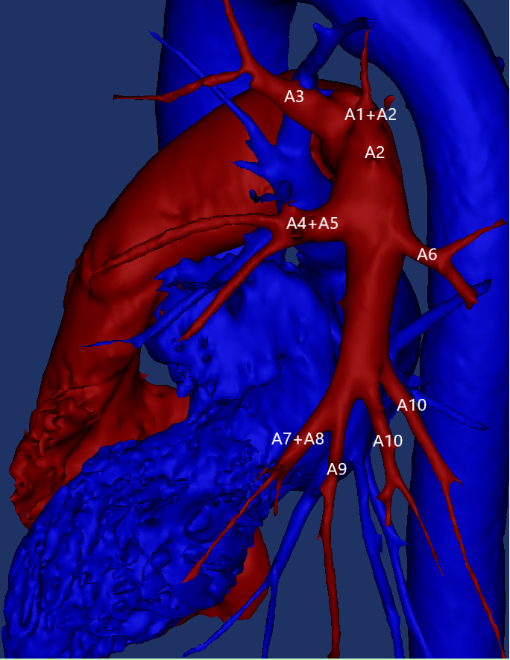


Figure 20: Right superior lobe of lung：A1+A2：Posterior apical artery ，A3：Anterior segmental artery（A1+A2 and A3 have the same trunk）。

Middle lobe of right lung：A4:Lateral segmental artery of middle lobe,A5:Medial segmental artery of middle lobe。

Right lower lobe of lung：A6：Inferior lobe superior segment artery，A7+A8：Trunk of anterior and medial basilar artery，A9+A10：Trunk of lateral and posterior basilar artery。

Figure 19：Left superior lobe of lung：A3：Anterior segmental artery，A1+A2：Posterior apical artery，A2：Anterior segmental artery，A4：Superior lingual artery，A5：Inferior lingual artery。

Left lower lobe of lung：A6：Inferior lobe superior segment artery，A7+A8+A9：Trunk of anterior and medial basilar artery，A10：Posterior basilar segment artery。

Figure 18：Right superior lobe of lung：A1：Apical segmental artery，A2：Posterior segmental artery，A3：Anterior segmental artery，A2+A3：Posterior segmental + Anterior segmental artery。

Middle lobe of right lung：A4:Lateral segmental artery of middle lobe,A5:Medial segmental artery of middle lobe。

Right lower lobe of lung：A6：Inferior lobe superior segment artery，A7+A8：Trunk of anterior and medial basilar artery，A9+A10：Trunk of lateral and posterior basilar artery。

Figure 17：Left superior lobe of lung：A3：Anterior segmental artery，A1+A2：Posterior apical artery，A2：Posterior segmental artery，A4+A5：Superior lingual artery and inferior lingual artery have the same trunk。

Left lower lobe of lung：A6：Inferior lobe superior segment artery，A7+A8：Trunk of anterior and medial basilar artery，A9：Lateral basal segment artery，A10：Posterior basilar segment artery。


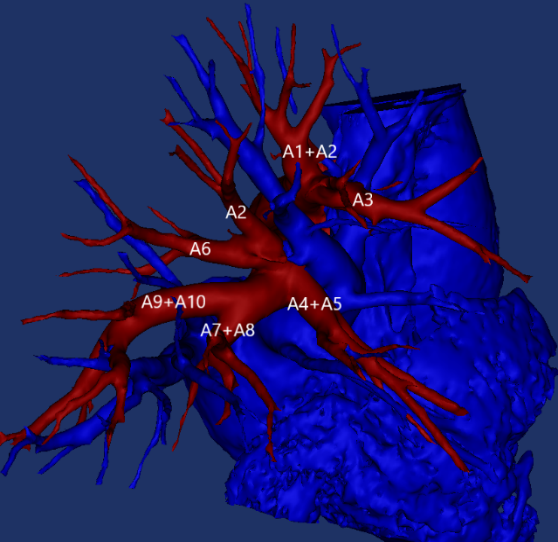

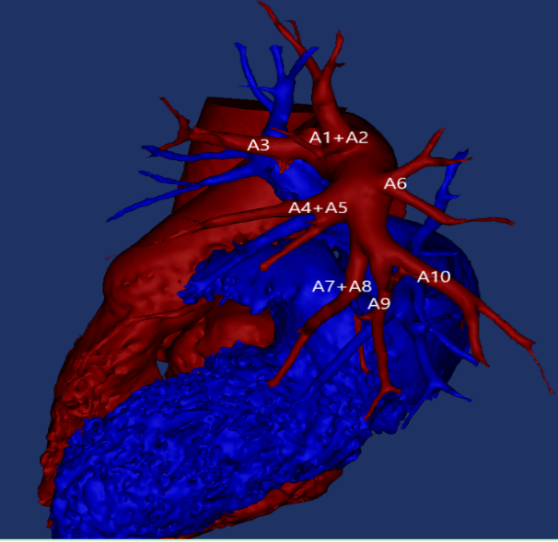

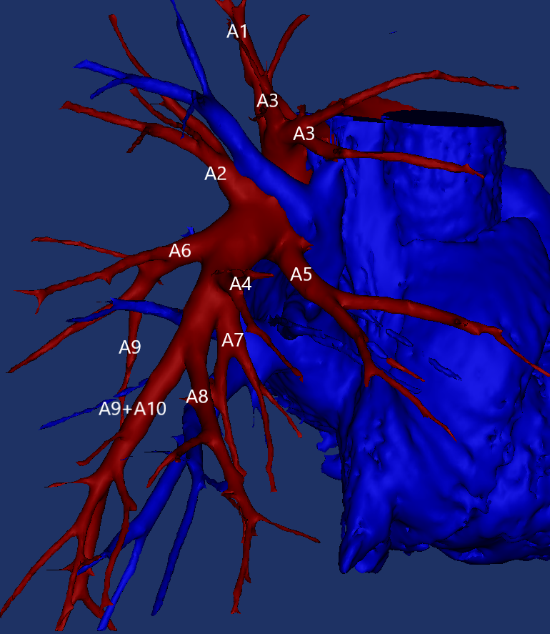

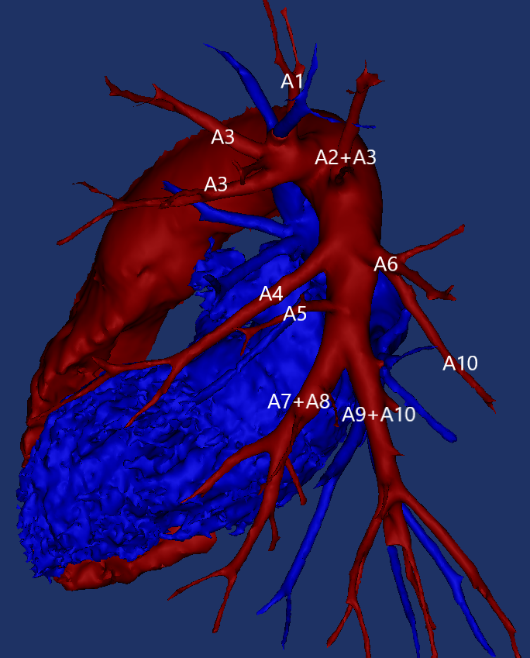


Figure 21：Left superior lobe of lung：A3：Anterior segmental artery，A1：Apical segmental artery，A2+A3：Posterior segmental + Anterior segmental artery，A4：Superior lingual artery，A5：Inferior lingual artery。

Left lower lobe of lung：A6：Inferior lobe superior segment artery，A7+A8：Trunk of anterior and medial basilar artery，A9+A10：Trunk of lateral and posterior basilar artery，A10：Posterior basilar segment artery。

Figure 22: Right superior lobe of lung：A1：Apical segmental artery， A3：Anterior segmental artery（A1 and two branches of A3 have the same trunk），A2：Posterior segmental artery。

Middle lobe of right lung：A4:Lateral segmental artery of middle lobe,A5:Medial segmental artery of middle lobe。

Right lower lobe of lung：A6：Inferior lobe superior segment artery，A7：Medial basal segment artery，A8：Anterior basilar segment artery，A9：Lateral basal segment artery，A9+A10：Trunk of lateral and posterior basilar artery。

Figure 24: Right superior lobe of lung：A1+A2：Posterior apical artery ，A3：Anterior segmental artery（A1+A2 and A3 have the same trunk），A2：Posterior segmental artery（A2 and A6 have the same trunk）。

Middle lobe of right lung：Middle Lobar Artery (dividing into medial and lateral segmental arteries)。

Right lower lobe of lung：A6：Inferior lobe superior segment artery（A2 and A6 have the same trunk），A7+A8：Trunk of anterior and medial basilar artery，A9+A10：Trunk of lateral and posterior basilar artery。

Figure 23：Left superior lobe of lung：A3：Anterior segmental artery，A1+A2：Posterior apical artery，A4+A5：Superior lingual artery + Inferior lingual artery have the same trunk。

Left lower lobe of lung：A6：Inferior lobe superior segment artery，A7+A8：Trunk of anterior and medial basilar artery，A9：Lateral basal segment artery，A10：Posterior basilar segment artery。


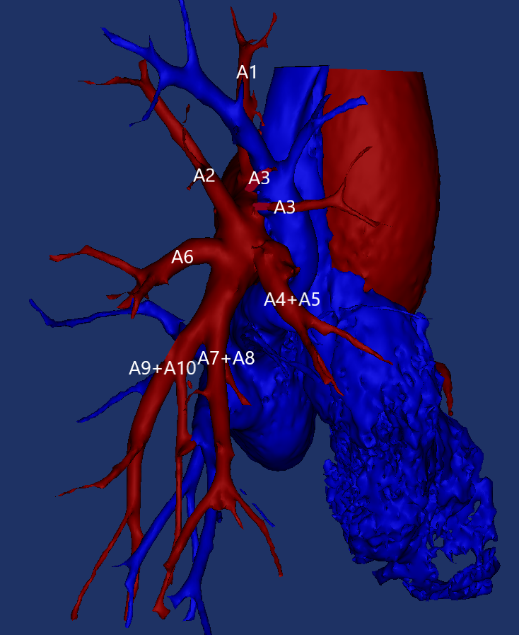

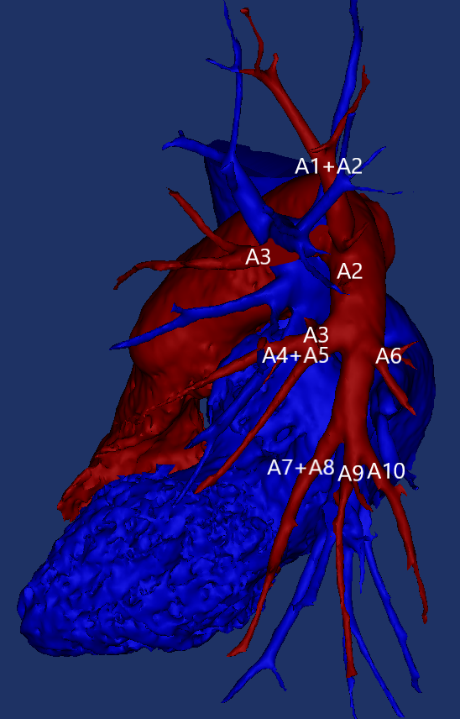

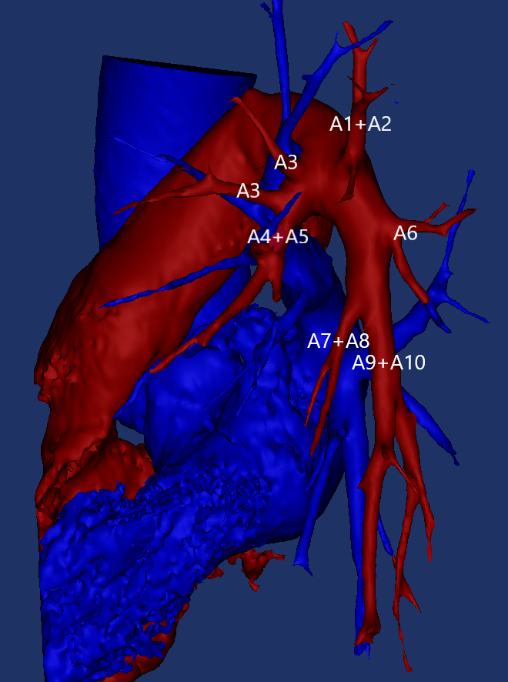


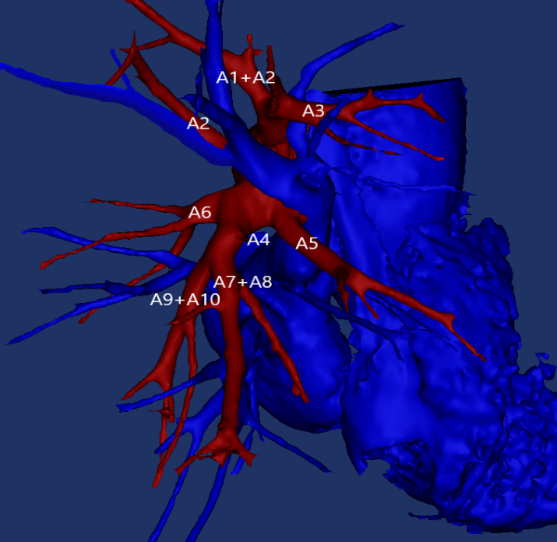


Figure 28: Right superior lobe of lung：A1+A2：Posterior apical artery ，A3：Anterior segmental artery（A1+A2 and A3 have the same trunk），A2：Posterior segmental artery（A2 and A6 have the same trunk）。

Middle lobe of right lung：A4:Lateral segmental artery of middle lobe,A5:Medial segmental artery of middle lobe。

Right lower lobe of lung：A6：Inferior lobe superior segment artery，A7+A8：Trunk of anterior and medial basilar artery，A9+A10：Trunk of lateral and posterior basilar artery。

Figure 27：Left superior lobe of lung：A3：Anterior segmental artery，A1+A2：Posterior apical artery，A2：Posterior segmental artery，A4+A5：Superior lingual artery + Inferior lingual artery have the same trunk。

Left lower lobe of lung：A6：Inferior lobe superior segment artery，A7+A8：Trunk of anterior and medial basilar artery，A9：Lateral basal segment artery，A10：Posterior basilar segment artery。

Figure 26: Right superior lobe of lung：A1：Apical segmental artery，A3：Anterior segmental artery（A1 and two branches of A3 have the same trunk），A2：Posterior segmental artery（A2 and A6 have the same trunk）。

Middle lobe of right lung：A4+A5：Middle Lobar Artery (dividing into medial and lateral segmental arteries)。

Right lower lobe of lung：A6：Inferior lobe superior segment artery，A7+A8：Trunk of anterior and medial basilar artery，A9+A10：Trunk of lateral and posterior basilar artery。

Figure 25：Left superior lobe of lung：A3：Anterior segmental artery，A1+A2：Posterior apical artery，A4+A5：Superior lingual artery + Inferior lingual artery have the same trunk（A3 and A4+A5 have the same trunk）。

Left lower lobe of lung：A6：Inferior lobe superior segment artery，A7+A8：Trunk of anterior and medial basilar artery，A9+A10：Trunk of lateral and posterior basilar artery。
